# Effect of cell retention techniques in *Komagataella phaffii* lab‐scale continuous processes

**DOI:** 10.1002/btpr.70092

**Published:** 2025-11-18

**Authors:** Marina Y. Linova, Satish K. Kodiripaka, Edite Martins, Sobhana A. Sripada, Stefano Menegatti, John M. Woodley

**Affiliations:** ^1^ Department of Chemical and Biochemical Engineering Technical University of Denmark Kongens Lyngby Denmark; ^2^ Department of Chemical and Biomolecular Engineering North Carolina State University Raleigh North Carolina USA; ^3^ Biomanufacturing Training and Education Center (BTEC) Raleigh North Carolina USA

**Keywords:** acoustic cell separation, cell retention, continuous bioprocessing, *Komagataella phaffii*, perfusion, protein production, vibrating membrane filtration

## Abstract

Perfusion technologies play a growing role in the implementation of continuous processes for biotherapeutics production in mammalian‐based manufacturing. However, their application to alternative production hosts is limited. Cell retention systems are of key importance for the efficiency of perfusion bioreactors. In this study, we investigate two cell retention technologies for the development of lab‐scale *Komagataella phaffii* continuous processes. An acoustic‐based process (AP) and a membrane‐based process (MP) were developed using an acoustic cell separator (ACS) and a vibrating membrane filtration (VMF) device, respectively. Both systems allowed for continuous cell recycle and production of scFv13R4 antibody fragment for 8 days (AP) and 9 days (MP), without loss in productivity, while maintaining high viability (greater than 90%). Higher volumetric and specific productivities were achieved during the AP process, namely 50.63 ± 1.63 mg L^−1^ day^−1^ and 1.09 ± 0.07 mg g^−1^ day^−1^, against the 32.29 ± 1.21 mg L^−1^ day^−1^ and 0.44 ± 0.02 mg g^−1^ day^−1^ afforded by the MP process. The VMF device provided 100% separation efficiency with biomass accumulating up to concentrations of 74.1 ± 0.1 g L^−1^ dry cell weight (DCW), whereas the acoustic device reached 55.1 ± 0.47 g L^−1^ DCW at 98% separation efficiency. The acoustic device showed selectivity towards larger and more complex cells in the yeast population, which might be linked to the observed higher productivities for the AP process. This study discusses the advantages and drawbacks of both cell retention technologies and provides an outlook towards their future investigation in *K. phaffii* perfusion processes.

## 1 INTRODUCTION

The biopharmaceuticals market is rapidly expanding towards new therapeutic modalities and product designs.^1^ Advanced protein therapeutics are becoming increasingly important, yet still not widely accessible due to high costs.^2^ The development of new protein production platforms using alternative hosts has been suggested as a viable solution to some of the economic and technical challenges faced by mammalian‐based processes.^3^, ^4^, ^5^ *Komagataella phaffii* (*Pichia pastoris*) is among the most researched non‐conventional hosts and is often suggested as a potential disruptor of current biomanufacturing processes.^6^ The interest in *K. phaffii* has led to the development of new and improved strains and the expression of many proteins.^5^, ^6^, ^7^, ^8^, ^9^, ^10^, ^11^, ^12^ This host has already been adopted in the commercial production of several recombinantly produced therapeutic proteins,^1^, ^13^ including the first approved aglycosylated monoclonal antibody.^14^ The first‐in‐human phase I clinical trial of a *K. phaffii*‐produced bispecific NANOBODY® was also recently reported.^15^ Some well‐known and desired biomanufacturing traits of *K. phaffii* include rapid growth to high cell densities, fewer secreted host cell proteins, and eliminating the need for viral clearance.

Fed‐batch is the primary mode of operation in high‐cell‐density *K. phaffii* cultivations.^16^ A typical process consists of three phases: an initial glycerol batch culture, followed by a glycerol fed‐batch phase, aimed at rapid biomass accumulation, and lastly a fed‐batch protein production phase using methanol as a sole carbon source. The tightly regulated alcohol oxidase promoter (P_*AOX1*_) is widely used for protein induction with *K. phaffii*. It is strongly induced by methanol but repressed by other types of carbon sources such as glycerol and glucose. Maximum specific growth rates (*μ*_max_) are lower on methanol in comparison to glycerol, and a further decrease is observed due to the burden posed by recombinant protein production. Additionally, strains with different methanol utilization types (Mut), defined by the deletion of *AOX1* and *AOX2* genes, also differ with respect to growth kinetics. *μ*_max_ between 0.02 and 0.16 h^−1^ is reported for Mut^+^ systems,^16^, ^17^ whereas significantly lower values, 0.011–0.035 h^−1^, describe Mut^S^ strains lacking the *AOX1* gene.^16^ In Mut^−^ systems, characterized by the deletion of both *AOX1* and *AOX2*, protein production is even occurring in an almost growth‐decoupled way.^18^ Even though continuous chemostat cultures are also investigated,^17^, ^19^ dilution rates are limited by *μ*_max_ and the cytotoxic effect of methanol above critical concentrations. Therefore, chemostat as an operational strategy is restrictive with respect to harvest rates and is less suitable for strains exhibiting slow growth or growth‐independent production kinetics. Furthermore, in continuous chemostat processes, the biomass present in the harvest stream must still be removed by a subsequent unit operation. In contrast, continuous perfusion processes employ cell retention allowing for a continuous outflow of cell‐free harvest. Initially investigated for the recovery of unstable proteins and the achievement of higher cell densities in mammalian cell culture, this mode of operation is suitable for the continuous recovery of product, despite the host's growth kinetics. Current interest in perfusion processes is also driven by the need for cost reduction, smaller facility footprint, and compatibility with continuous downstream strategies.^20^, ^21^

Robust cell separation is a prerequisite for a successful perfusion process. Existing cell retention techniques, used in mammalian perfusion processes, fall into several main groups: filters, continuous centrifuges, gravity settlers, and acoustic wave separators.^22^, ^23^, ^24^ Each cell retention technology is developed based on one or several cell‐specific attributes, such as size, density or compressibility, and each possesses certain advantages and drawbacks. Nowadays, tangential flow filtration (TFF) and alternating tangential flow filtration (ATF) are the most used systems in perfusion upstream processes.^25^

Despite the many parallels between yeast and mammalian cell‐based processes, intrinsic differences exist between the two hosts, which warrant special considerations when developing perfusion processes. In comparison to mammalian cells, yeasts are smaller (3–5 μm), feature a spherical to ellipsoidal shape, expand by cell budding, and present lower sensitivity to shear stress.^26^ Furthermore, *K. phaffii* upstream processes are typically operated at higher biomass concentrations and present a high oxygen demand due to the nature of methanol utilization. However, over the past two decades, technological development has mainly addressed the needs and challenges of mammalian production hosts. That raises the question of whether some of the currently available cell separation technologies are suitable for the development of *K. phaffii* perfusion processes. Although the concept of using cell recycle for high‐cell‐density *K. phaffii* cultures has been suggested as an alternative option to batch and fed‐batch processes more than 20 years ago,^27^, ^28^ investigations focusing on *K. phaffii* perfusion processes at bioreactor scale remain rare.^29^, ^30^ Some examples of perfusion microdevices for *K. phaffii* have also been reported.^31^, ^32^ Addressing this gap, in the present study we evaluate the performance of two distinct technologies for their potential use as cell retention devices in high‐cell‐density *K. phaffii* perfusion processes, namely an acoustic cell separator (ACS) and a vibrating membrane filtration (VMF) device. VMF builds on the principle of tangential flow filtration by improving fouling mitigation through the vibration of a membrane relative to the cell suspension feed.^33^ In contrast, the acoustic cell separator belongs to a group of cell retention devices that do not rely on a physical barrier for separation, as the cells are retained in the pressure nodes of a created acoustic field.^34^ Hence, this technology is not prone to fouling but typically operates at lower separation efficiencies.^35^

This study demonstrates the application of VMF and ACS for continuous cell recycling in high‐density *K. phaffii* cultivations. The developed processes enable a comparison of the performance and efficiency of these two cell retention techniques across increasing biomass concentrations. Furthermore, their impact on cell viability, heterogeneity, and recombinant protein production is assessed. This work establishes a foundation for the future development of *K. phaffii* steady‐state perfusion processes. For simplicity, we will abbreviate the process using VMF as the membrane‐based process (MP) and the process using ACS as the acoustic‐based process (AP).

## 2 MATERIALS AND METHODS

### 2.1 Bioreactor cultivations

*Strain and inoculum preparation. Komagataella phaffii* (*Pichia pastoris*) X33 Mut^+^ was modified for the secretion of a 6x His‐tagged single‐chain variable antibody fragment, scFv13R4, under the control of the tightly regulated P_*AOX1*_. The development of the strain has been described in prior work.^36^ Three 100 mL BMGY shake flasks were inoculated with fresh colonies from a YPD agar plate and grown overnight at 30°C and 250 rpm. To prepare the final inoculum, the BMGY overnight cultures were concentrated, washed, and resuspended in BFM21 defined cultivation medium. The composition of BFM21 and the trace metal solution (PTM4) used here was as previously described,^36^, ^37^ and listed in Table S1.

*Cultivation conditions*. Small‐scale cultivations were carried out in an Applikon miniBio bioreactor (Getinge Applikon, The Netherlands) controlled using my‐Control controller (Getinge Applikon, The Netherlands) and Lucullus software (Securecell AG, Switzerland). Cell recycle during protein production was achieved by using either VMF or ACS as a cell retention technique. BFM21 with PTM4 supplemented with either glycerol or methanol was used throughout the cultivation. The temperature was maintained at 28°C and the addition of 28%–30% (w/w) ammonia solution and 85%–90% (w/w) phosphoric acid was used to control the pH at 5 and 5.5 during the biomass accumulation and protein production phases, respectively. The bioreactor was aerated at 300 mL min^−1^ and the stirrer speed was constant at 1200 rpm. The addition of pure oxygen gas was triggered by a drop in dissolved oxygen (DO) values below 50% and limited to a maximum of one‐third of the total gas flow.

*Biomass accumulation phase*. The biomass accumulation phase comprised a glycerol batch, followed by a glycerol fed‐batch phase. The initial working volume of the bioreactors was 300 mL BFM21 with 4% (v/v) glycerol and 7.5 mL L^−1^ PTM4. The bioreactors were inoculated at a starting cell density of OD600 ~5. Upon the end of the glycerol batch phase, indicated by a spike in DO, 40% (v/v) glycerol solution supplemented with 12 mL L^−1^ PTM4 was fed to the bioreactor at 0.05 mL min^−1^ until OD600 reached ~100. The reactor volume at the end of the glycerol fed‐batch was 317 and 320 mL for the VMF and ACS setup, respectively.

**FIGURE 1:**
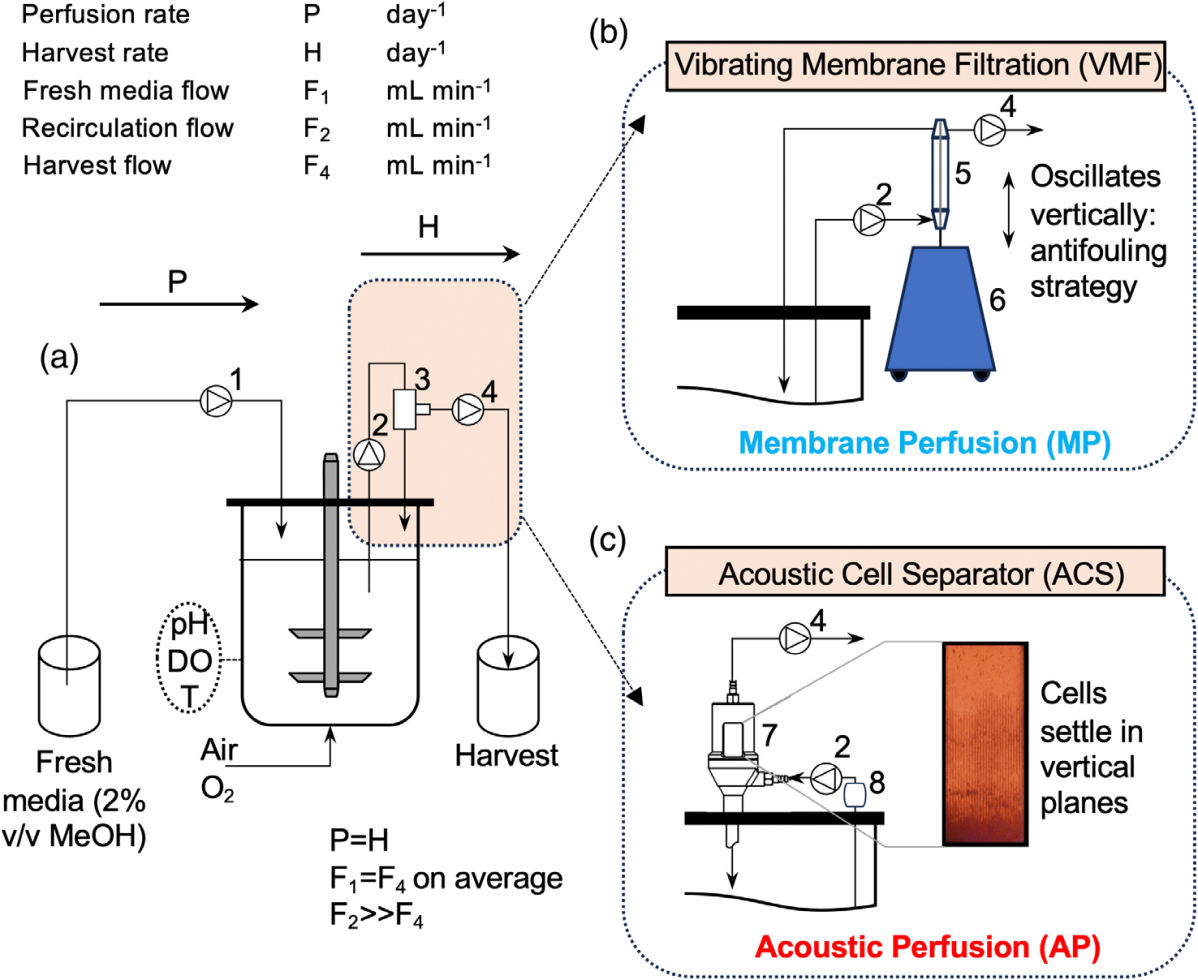
Schematic of the experimental set‐up used for perfusion‐based cultivations of *Komagataella phaffii*. (a) General schematic of perfusion reactor set‐up. (1) Feed pump, supplying fresh media at *F*_1_; (2) Recirculation pump, operating at *F*_2_; (3) Cell retention device; (4) Harvest pump, operating at *F*_4_; Cell suspension from the reactor is supplied to the cell retention device at a flow rate *F*_2_ much greater than the harvest flow rate *F*_4_, ensuring a stable harvest stream. The clarified stream is continuously collected at harvest rate *H*, while a concentrated cell stream is returned from the cell retention device back to the bioreactor. As no bleed stream is used in the setup, fresh media was added to the bioreactor at *P* = *H*, such that the reactor volume is in average constant, by adjusting *F*_1_ to match, on average, *F*_4_. (b) Schematic of cell retention via vibrating membrane filtration (VMF). (5) Membrane module, hosting a 0.2 μm membrane. (6) Drive unit, ensuring vertical oscillation of the membrane module. (c) Schematic of cell retention via acoustic cell separator (ACS). (7) Acoustic chamber; (8) air trap, consisting of additional peristaltic pump and a glass container. (b and c) are operated as described in (a).

*Protein production phase*. Once the desired cell density was reached, a continuous cell recycle was initiated, and the process was switched to a perfusion‐based mode of operation using methanol as protein inducer and the sole carbon source. A general description of the experimental set‐up is schematically presented in Figure 1a. The continuous flow of fresh cultivation media (BFM21 with 12 mL L^−1^ PTM4) containing 2% (v/v) MeOH was initiated by the built‐in pumps of the bioreactor controller and supplied at 0.34 ± 0.01 and 0.33 ± 0.01 mL min^−1^ throughout the processes, for VMF and ACS, respectively. Cell suspension was pumped from the bioreactor to the cell retention devices at 5 mL min^−1^ and then split into a clarified harvest collected at 0.312 and 0.309 mL min^−1^ (VMF and ACS, respectively), and a cell stream recycled back to the bioreactor. The processes were operated at perfusion rates P between 1.2 and 1.4 day^−1^. The perfusion rate P defines the rate at which fresh medium is supplied while spent medium is removed. It is expressed as the sum of the harvest rate H and cell bleed rate B:

(1) P=B+H.

As there was no dedicated stream for cell bleed in either of the configurations (*B* = 0), P equals the volume of harvested media $V_{\text{daily harvest}}$ per bioreactor volume $V_{\text{reactor}}$ per day (vvd, or day^−1^):

(2) $$P=H=V_{\text{daily harvest}}/V_{\text{reactor}}.$$

The clarified harvest (perfusate) was collected at a constant flow rate, resulting in a constant volume of daily harvested media $V_{\text{daily harvest}}$. The bioreactor volume $V_{\text{reactor}}$ reflects the cell suspension volume in the bioreactor vessel itself, together with that in the cell retention system. The fresh media flow rate was used to control the desired vvd by level‐based adjustment of $V_{\text{reactor}}$. Any fluctuations in the volume due to changes in the fresh media flow rate as well as the differences in the hold‐up volumes of the two cell retention devices were reflected in $V_{\text{reactor}}$. Protein production in perfusion‐based mode proceeded for 209 and 194 h for the membrane and acoustic process, respectively.

*Cell retention via VMF*. The cell retention driven by vibrating membrane filtration is schematically depicted in Figure 1b. A benchtop membrane filtration unit Vibro‐LabEV (SANI Membranes, Denmark), consisting of a Vibro Lab35 membrane module equipped with a 0.2 μm GVPP membrane (membrane area of 35 cm^2^) and a Vibro Lab‐VE drive was used for cell separation. Cell suspension was fed to an inlet at the bottom of the membrane cartridge. Clarified harvest was collected from the top using the permeate port of the cartridge, while the remaining cell‐containing stream exiting through the retentate port was recycled back to the reactor. The Vibro Lab‐VE drive unit oscillates vertically, applying turbulence to the membrane and acting as an anti‐fouling mechanism.

*Cell retention via ACS*. The acoustic cell retention, schematically shown in Figure 1c, was implemented using an Applikon mini Biosep acoustic cell retention device (Getinge Applikon, The Netherlands) consisting of a resonator chamber and a controller. The controller provides a high‐frequency voltage signal generating a resonant standing wave field in the chamber. The resonance chamber, with a volume of 1.5 mL, was mounted on the headplate of the bioreactor and connected to the controller, setting the strength of the acoustic field (1 W), frequency (2.1 MHz), ON time (5 min), and OFF time (3 s). Cell suspension was fed to the bottom of the acoustic chamber and recirculated using an external peristaltic pump. Clarified harvest was drawn from the top of the chamber during ON time (active acoustic field and cell retention) by an external peristaltic pump. The remaining cell suspension stream was recycled back to the reactor from the bottom of the chamber. During OFF times, no harvest is collected, and cells previously retained in the acoustic chamber flow back to the bioreactor before the next ON period begins. An air trap was included before the circulation pump to prevent air bubbles from entering the chamber and disturbing the field.

### 2.2 Biomass concentration

Biomass concentration was monitored at the beginning of each cultivation phase (glycerol batch, glycerol fed‐batch, and production phase), and approximately every 24 h during the protein production phase. Cell growth was monitored by optical density at 600 nm (OD600) and dry cell weight (DCW). OD600 was measured using UV1800 UV‐spectrophotometer (Shimadzu, Japan). Dry cell weight measurements were performed in technical triplicates. The samples were centrifuged at 10000×*g* for 5 min and dried at 50°C. For the acoustic process, the biomass concentration in the perfusate was also analyzed, by OD600 measurements only. Separation efficiency was calculated based on the ratio between the concentration of cells in the reactor $C_{\text{reactor}}$ and the harvest $C_{\text{harvest}}$:

(3) $$\text{Separation efficiency}=1-C_{\text{harvest}}/C_{\text{reactor}}.$$

### 2.3 HPLC methanol determination

The harvest stream (perfusate) was sampled daily for methanol quantification. The concentration of methanol in the perfusate was measured by HPLC (Ultimate 3000, Thermo Scientific, USA) using an Aminex HPX‐87H column (Bio‐Rad Laboratories, Inc., USA). A refractive index (RI) detector was used to quantify methanol (4 mM H_2_SO_4_ mobile phase at 0.6 mL h.^−1^ at 60°C). The samples were conditioned by using 10% (v/v) mobile phase and then filtered through 0.45 μm filters. Cultivations were performed under methanol‐limiting conditions. A standard curve in the range of 0.2%–3% (v/v) was used to confirm the absence of methanol in the collected perfusate.

### 2.4 Protein analysis

The harvest stream (perfusate) was sampled daily for scFv13R4 and HCPs quantification. Additionally, the supernatant of samples taken from the bioreactor for determining biomass concentration was saved and used for scFv13R4 quantification. Bioreactor and harvest samples, together with in‐house purified scFv13R4 standards at different concentrations (10, 20, 40, and 80 mg L^−1^), were mixed with NuPAGE™ LDS Sample Buffer (4x) (Invitrogen, Thermo Fisher Scientific), and incubated at 70°C for 10 min. A Precision Plus Protein™ Standard (Catalog #161‐0363, Bio‐Rad Laboratories) served as the molecular weight standard. The samples were separated on a gradient NuPAGE™ 4%–12% Bis‐Tris gel (Invitrogen, Thermo Fisher Scientific) for 38 min at a constant voltage of 200 V using MES running buffer (Invitrogen, Thermo Fisher Scientific). After the electrophoresis, the gels were washed with deionized water and stained for 1 h with a Coomassie Blue‐based stain (Invitrogen, Thermo Fisher Scientific). After distaining, the gels were imaged on the GelDoc Go Gel Imaging System (Bio‐Rad Laboratories), and densitometry analysis was performed using Image Lab Software. The scFv13R4 concentration was measured in technical triplicates and calculated based on a standard curve generated from the purified scFv13R4 standards. The HCP titers were quantified in duplicates by the *Pichia pastoris* Host Cell Proteins 2nd Generation ELISA kit (Cygnus Technologies), following the manufacturer's protocol.

### 2.5 Evaluation of productivity

The productivity of the processes is evaluated based on volumetric productivity $Q_{\text{scFv}}$ and specific productivity $q_{\text{scFv}}$, determined as:

(4) $$Q_{\text{scFv},i}=c_{\text{scFv},i}\times P_{i}=X_{i}\times q_{\text{scFv},i},$$

(5) $$q_{\text{scFv},i}=\frac{c_{\mathrm{scFv},i}\times P_{i}}{X_{i}}=\frac{Q_{\text{scFv},i}}{X_{i}},$$

where $c_{\mathrm{scFv},i}$ and $X_{i}$ are product titer and biomass concentration from dry weight measurements, at a given time, respectively.

The relationship between $q_{\text{scFv},i}$ and the specific methanol consumption rate $\nu_{\text{MeOH}}$ is investigated. $\nu_{\text{MeOH}}$ is determined as:

(6) $$\nu_{\text{MeOH},i}=\frac{P_{i}\times \left(C_{\text{feed}}-C_{\text{harvest}}\right)}{X_{i}},$$

where $P_{i}\times \left(C_{\text{feed}}-C_{\text{harvest}}\right)$ is the volumetric consumption rate, and $C_{\text{feed}}$ and $C_{\text{harvest}}$ are the methanol concentration in the feed media and perfusate, respectively.

The specific rate of HCP expression $q_{\mathrm{HCP},i}$ was calculated similarly to $q_{\text{scFv},i}$, but considering the HCP concentration defined as $c_{\mathrm{HCP},i}$ at a given time:

(7) $$q_{\mathrm{HCP},i}=\frac{c_{\mathrm{HCP},i}\times P_{i}}{X_{i}}=\frac{Q_{HCP,i}}{X_{i}}.$$

### 2.6 Flow cytometry

Flow cytometry analysis was performed every other day. Samples were prepared by dilution with PBS buffer to an OD600 ~1, followed by 2× washes with PBS buffer. Propidium iodide (PI)‐positive control samples were incubated for 10 min in an 80°C water bath. Samples were stained using PI stain (20 mM) provided as part of the FungaLight™ CFDA, AM/Propidium Iodide Yeast Vitality Kit (Molecular Probes™, Invitrogen, Thermo Fisher Scientific). The final concentration of PI in the sample was 20 μM. Flow cytometry was performed using CytoFLEX (Beckman Coulter, USA) at 10 μL mL^−1^ for 5 μL. Data acquisition and analysis were done by CytExpert 2.4 (Beckman Coulter, USA). Cells were gated for forward and side scatter, and forward scatter height and width. PI‐stained controls were also gated for FSC and fluorescent intensity. PI‐fluorescent intensity was read using a 610/20 bandpass filter. Test samples stained with PI, and PI‐positive control were compared to determine the degree of cell damage. A 488 nm laser was used for excitation.

## 3 RESULTS AND DISCUSSION

The choice of cell retention devices in this study was driven by the need for technologies that can withstand high cell densities, without significant fouling, and the commercial availability of devices that can operate at perfusion rates suitable for small‐scale (~320 mL) systems. Two experiments were conducted with each of the chosen cell retention devices: MP1 and MP2 which correspond to membrane‐based processes using a VMF device; AP1 and AP2 which correspond to acoustic‐based processes performed with an ACS device. Processes MP1 and AP1 were shorter than MP2 and AP2. The remaining operating conditions were the same for all four processes. While there are no differences in the experimental set‐up for MP1 and MP2, an air trap was introduced to the experimental set‐up used for AP2 in order to improve cell separation efficiencies. Data from all four processes have been used to describe the performance of the cell retention devices with respect to separation efficiencies, and their impact on cell heterogeneity. Membrane integrity, HCP levels and scFv13R4 production are evaluated only for processes MP2 and AP2, due to the extended process times. This study is the first to demonstrate the application of VMF and ACS in yeast perfusion processes for recombinant protein production.

### 3.1 Cell separation performance of VMF and ACS devices

Since the development of cell retention devices for perfusion processes has been focused on mammalian cell culture, ATF and TFF devices with different fouling mitigation strategies have found the broadest application.^24^, ^25^, ^38^ However, *K. phaffii* processes are operated at more than 100‐fold higher biomass concentrations than CHO cells, the most utilized mammalian expression host.^16^, ^39^ Therefore, cell retention technologies with a low risk of fouling are essential for *K. phaffii* perfusion systems.

**FIGURE 2:**
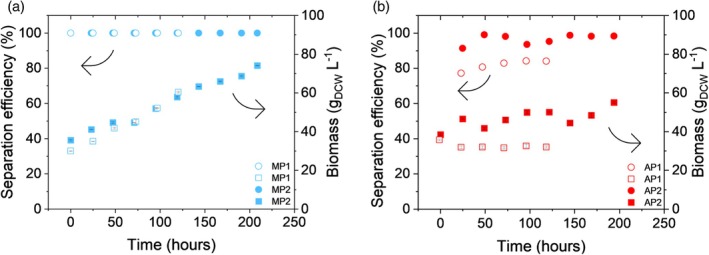
Time course of biomass accumulation (squares) and separation efficiencies of the cell retention devices (circles) in *Komagataella phaffii* cultivations after the initiation of cell recycle. (a, b) Biomass concentrations are from technical triplicates of DCW measurements. Error bars represent standard deviations. (a) Evaluation of VMF in MP1 (open) and MP2 (closed). No cells were detected in the perfusate stream, therefore 100% separation efficiency is assumed. (b) Evaluation of ACS in AP1 (open) and AP2 (closed). Separation efficiencies are calculated from single OD600 measurements.

Here we investigate two cell retention devices, VMF and ACS, both tackling the issue of fouling by relying on distinct technological concepts. VMF adds a dynamic element to crossflow filtration which acts as an antifouling mechanism in place of increasing crossflow velocities or backwashing which are commonly used approaches with TFF systems. Therefore, in comparison to classical TFF systems, VMF can operate at significantly higher biomass concentrations within a similar range of flux values.^40^ The VMF device used in our perfusion set‐up achieved 100% separation efficiency throughout the whole duration of both MP processes (Figure 2a). Biomass concentrations of 66.5 ± 0.5 and 74.1 ± 0.1 g L^−1^ were reached after 120 and 209 h for MP1 and MP2, respectively (Figure 2a). In contrast, cell bleed with the harvest stream is inevitable for acoustic‐based cell retention technologies, and as expected the cell separation efficiencies were lower for the AP in comparison to MP processes (Figure 2b). The highest recorded separation efficiency for AP1 was 84.2%, and the highest biomass concentration of 35.7 g L^−1^ was observed at the beginning of the process. Air bubbles entering the acoustic chamber from the recirculation line were disturbing the settling and retention of cells in the acoustic field. To counteract the disturbance, an air trap was introduced in the AP2 experimental set‐up. As a result, the separation efficiency was improved, with an average value of 96.6 ± 2.7%. Consequently, the cell density in the reactor was also improved, reaching 55.1 ± 0.47 g L^−1^ by the end of the process (Figure 2b). The key advantage of acoustic cell separators in comparison to filter‐based devices is the absence of a physical barrier. Instead, they rely on the generated acoustic field, resulting in foul‐free technology,^41^ although with the trade‐off of lower separation efficiencies. Cell separation efficiencies above 90% have been achieved with ACS for CHO perfusion processes.^35^, ^42^ Despite the substantial difference in cell densities, we achieved similar values in the AP2 process.

Although the biomass concentrations observed in both the MP and AP processes are within the investigated ranges for the expression host,^16^ methanol‐grown *K. phaffii* cultures can reach dry cell weight concentrations even above 100 g L^−1^ in fed‐batch processes. However, it is up to debate what cell densities are physiologically and practically relevant. Factors such as viability and cell‐specific productivity have a great impact on the final process yields and can be impacted by the cell density in the reactor. The results from the MP processes indicate that the capacity of the VMF device likely surpasses what was reached during our experiments, as a stable flux of 5.3 L m^−2^ h^−1^ was sustained for 209 h of operation. After 125 h of operation, the measured flow rate of the harvest pump decreased by approximately 4% in comparison to its setpoint, likely due to minor fouling events. The pump setpoint was increased accordingly to maintain the desired flow rate. Since VMF studies on yeast processes have been focused on batch cell harvest,^40^, ^43^ it is not feasible to establish a direct comparison with perfusion processes, where extended operation times and different harvest rates are desirable. Similarly, the application of acoustic cell retention in yeast processes has so far been limited to the preliminary demonstrations of the technology^44^ and more recently to intensify bioethanol production,^45^, ^46^ limiting the possibility of meaningful comparison for the performance of the device in the context of perfusion processes. Here we have demonstrated prolonged operation times and higher biomass concentrations than previously reported with ACS.

Similar trends emerge for both technologies with respect to their application in biopharmaceutical manufacturing throughout the years. The technical complexity of earlier VMF devices has been the main cause of their limited application,^47^ and is probably the reason why nowadays their use is mainly reported in wastewater treatment^48^, ^49^ and algae processes.^50^ However, advances in the field have led to the development of new VMF technology,^51^ as used in this study, and recently demonstrated for ultrafiltration during mRNA purification.^52^ Although many examples for the application of acoustic wave separators in mammalian perfusion processes exist, interest in this technology has substantially decreased over the past two decades.^24^ However, recent studies indicate a renewed attention towards the technology, namely for the production of viral vectors and viral particles.^53^, ^54^ Additionally, acoustic cell separators are nowadays available with a perfusion capacity of 1000 L day^−1^ (Getinge Applikon, The Netherlands), and scale up to 200 L day^−1^ at 96% separation efficiencies with CHO cells has been reported.^35^ Nevertheless, due to the remaining cells in the harvest stream, ACS‐based perfusion requires an additional filtration step, adding complexity to the overall process.

### 3.2 Characterization of cell heterogeneity using flow cytometry

**FIGURE 3:**
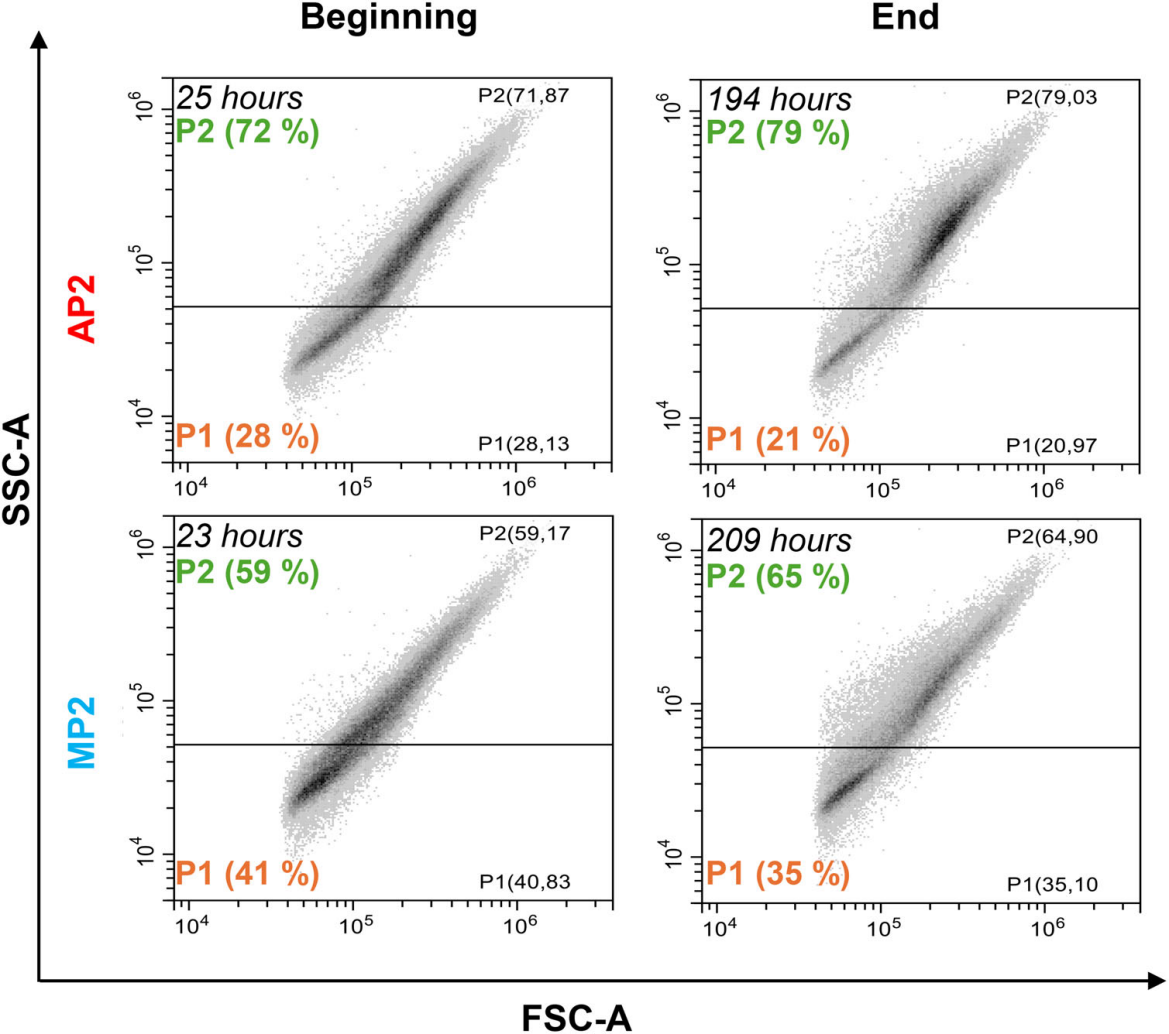
Representative FSC‐SSC density plots from the beginning and end points of processes MP2 (bottom) and AP2 (top). Two *Komagataella phaffii* sub‐populations are present: P1‐small size and low complexity (low FSC and SSC), and P2‐larger size and higher complexity (higher FSC and SSC).

Flow cytometry was used to characterize *K. phaffii* during the perfusion‐based processes. FSC and SSC data, relative to cell size and internal complexity, are used to describe the heterogeneity of the analyzed populations. Regardless of the cell retention device, FSC–SSC density plots show the presence of two subpopulations: P1, characterized by lower FSC and SSC values, indicating small cell size and low complexity; and P2, corresponding to higher FSC and SSC values, thus expected to describe larger and more complex cells (Figures 3 and S1).

**FIGURE 4:**
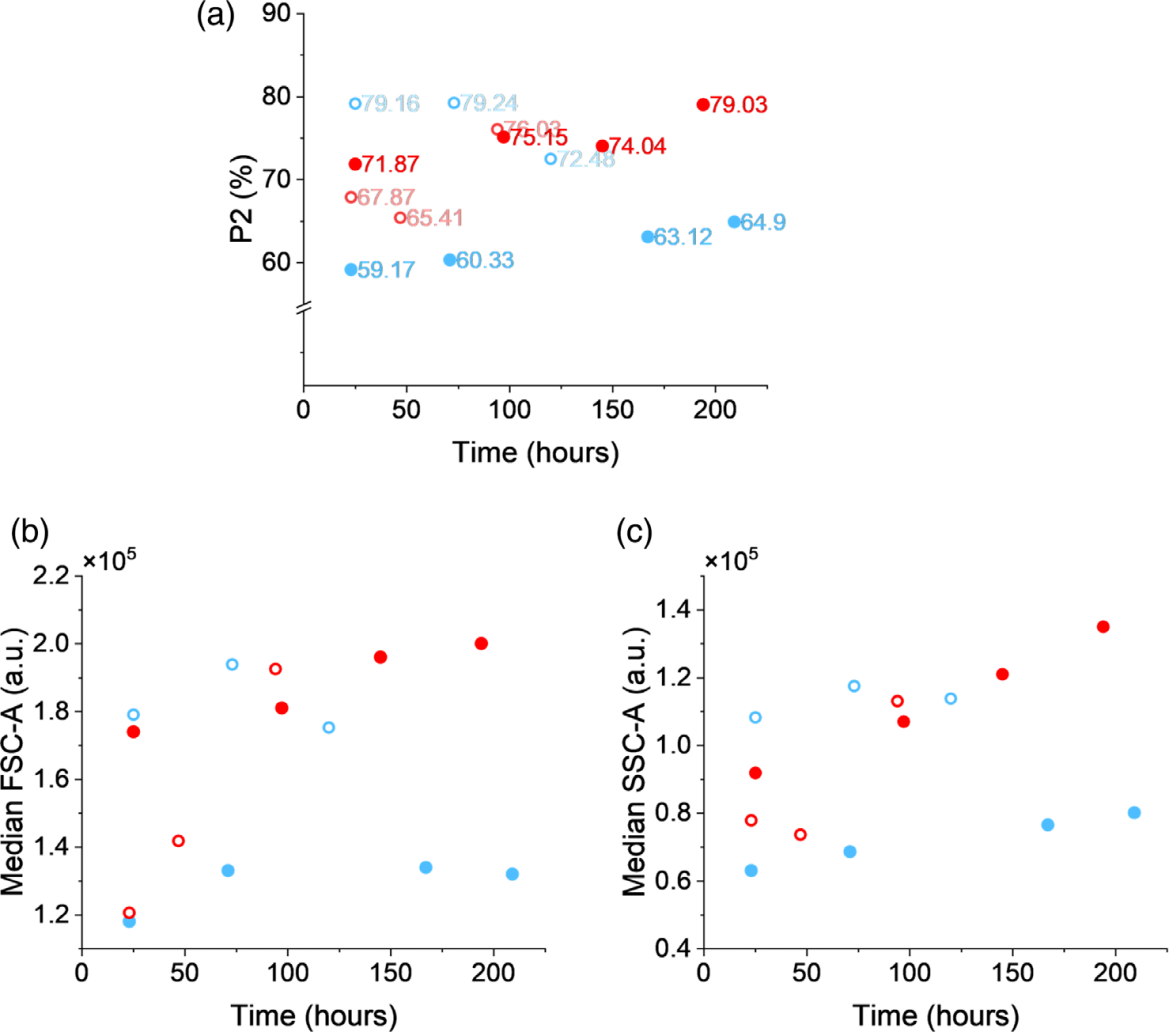
Changes in FSC and SSC over time across processes MP1, MP2, AP1 and AP2 after the initiation of cell recycle. MP processes are shown in blue, and AP processes are shown in red. MP1 and AP1 are represented by open, and MP2 and AP2 by closed symbols. (a) Changes in relative abundance of subpopulation P2. (b) Changes of median FSC of the overall *Komagataella phaffii* populations (P1 + P2). (c) Changes of median SSC of the overall populations (P1 + P2).

While in the beginning of the perfusion‐based process P1 and P2 seem merged; over time the two populations become more distinct. Except for process MP1, the relative abundance of P2 increases with the progression of the experiments (Figure 4a).

New yeast has the smallest, most uniform and therefore least complex cells, whereas budding introduces an increase in size and complexity. Furthermore, big oval cells can also be found in *K. phaffii* cultivations. Several sub‐populations have previously been identified through microscopic evaluation of shake flask cultures.^55^ Methanol metabolism itself leads to morphological changes and increased complexity in *K. phaffii* due to peroxisomal accumulation of AOX (alcohol oxidase), which catalyzes the oxidation of methanol. The enzyme forms large crystalloids stored in peroxisomes, which can occupy up to 80% of the cell in methylotrophic yeasts.^56^ Changes in mitochondrial size and number were also observed during methanol‐induced protein production.^57^

To obtain a more thorough understanding of how cell retention technologies influence the heterogeneity of *K. phaffii*, the changes in median FSC and SSC of the whole population (P1 + P2) are compared. The median values of FSC for both AP1 and AP2 show a clear upward trend in comparison to the MP processes, where the values for the parameter either plateau (MP2) or do not indicate either an upward or downward trend (MP1) (Figure 4b). All cells are retained in the reactor when VMF is used during MP processes. Therefore, when sub‐populations characterized by different cell sizes increase proportionally, a relatively unchanged median FSC is expected. On the other hand, such a proportional increase is less likely for AP processes, where cell bleed occurs with the harvest stream. The increase in particle size promotes the formation of aggregates at the pressure nodes of the generated acoustic standing wave, thus improving their retention.^58^ As a result, smaller particles and cells are preferentially purged out of the system. In comparison to AP2, cell retention in AP1 was worse and the separation efficiency lower (Figure 2). Therefore, the abundance of larger cells increased more rapidly, and was recorded as a steeper increase of median FSC in AP1 as compared to AP2, illustrating the cell size‐based selectivity phenomenon during AP processes. The associated advantages of such selectivity include the removal of cell particles, dying cells with reduced size due to loss of cell volume,^59^ and newly divided cells that lack the high amounts of AOX‐containing peroxisomes.^57^, ^60^ However, if the AP processes are not operated within suitable separation efficiencies, there is an increasing risk of bleeding cells adept at target protein expression, which would ultimately prove disadvantageous to the process yield.

In contrast to the median FSC, the median SSC is expected to increase for both process types. The accumulation of enzymes for methanol utilization, such as AOX, is needed in all viable cells and is associated with an increase in complexity. Whilst the specific methanol uptake rate and methanol utilization might differ between the present subpopulations in each process, a general trend of increasing cell complexity is to be expected for any process that uses methylotrophic *K. phaffii*. That was observed in all processes apart from MP1, where it was difficult to identify a defined trend (Figure 4c). Interestingly, AP1 and AP2's median SSC values were characterized by a steeper increase than MP2, where a more gradual increase is observed. As there is a positive correlation between size and complexity, the selectivity of ACS towards larger cells in AP processes will result in a higher abundance in comparison to MP processes where all cells are retained equally.

Notably, the median FSC and SSC values and the abundance of P2 were different among the four processes at similar times. The disparity is already evident after approximately 24 h of operating the processes in perfusion‐based mode. Therefore, other factors related to the biomass accumulation phase, and therefore unrelated to methanol metabolism and cell retention technologies, might have impacted heterogeneity even before cell recycle was initiated.

### 3.3 Cell membrane integrity

Maintaining high cell viability during *K. phaffii* perfusion‐based processes is of foremost importance for sustaining stable productivity over extended process times.^61^ Cell breakage can cause the release of cellular proteases, resulting in the degradation of the target product and a consequent decrease in process yield.^62^, ^63^ With the aim of identifying cell retention‐specific effects on viability, certain parameters that are associated with cell damage and are common to the two systems were kept the same for all processes. Continuous cell recirculation using peristaltic pumps is unavoidable for both systems but kept at the same rate of 5 mL min^−1^. Furthermore, methanol levels in the media feed (2 v/v%) were within an optimal range for maintaining viable *K. phaffii* cultivations with respect to methanol toxicity.^64^ Additionally, results from HPLC analysis of perfusate samples demonstrate that methanol was fully depleted (Figure S2). Notably, high cell density fed‐batch cultures are more prone to exhibit decreased viability due to the accumulation of toxic by‐products from incomplete methanol degradation in comparison to continuous processes that enable their removal together with the spent media.

**FIGURE 5:**
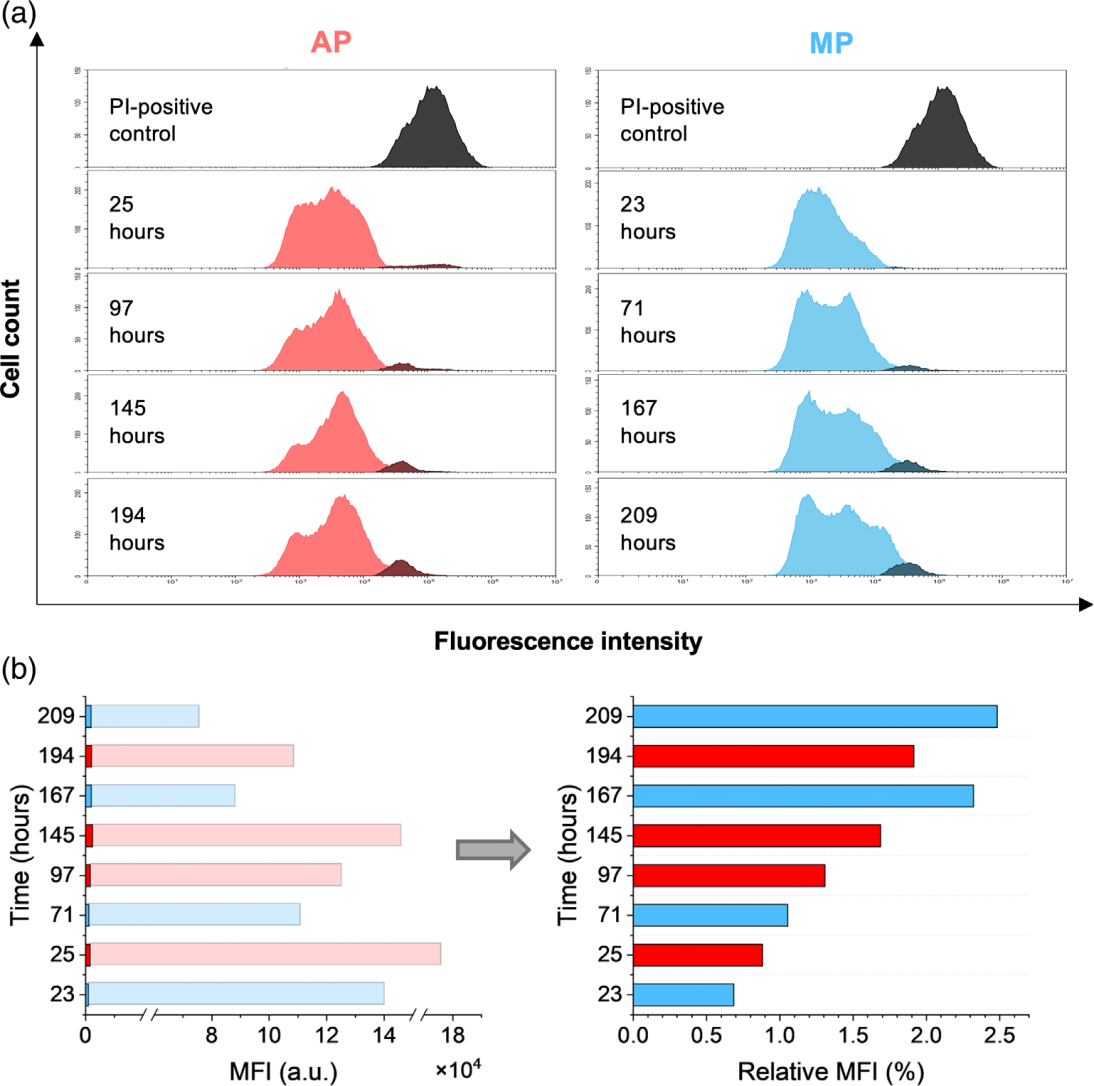
Flow cytometry assessment of *Komagataella phaffii*'s cell damage throughout processes MP2 (blue) and AP2 (red). The indicated time corresponds to the hours of operation in perfusion‐based mode. (a) Representative histograms of PI‐stained heat‐killed *K. phaffii* (PI‐positive control) on top, followed by histograms of PI‐stained samples taken throughout the two processes. The peaks overlapping with the PI‐positive control are highlighted in darker color. (b) Left: Bar plot showing the comparison between the median fluorescence intensity (MFI) of PI‐stained perfusion samples (darker‐colored bars) and heat‐killed PI‐control samples (lighter‐colored bars). Right: Bar plot of the calculated relative MFI (MFI of perfusion samples relative to control samples).

*K. phaffii*'s viability was evaluated using propidium iodide (PI) staining as an indicator of membrane integrity (Figure 5), a widely used method for assessing response to stress conditions in *K. phaffii* cultivations.^65^, ^66^ PI permeates cells with compromised membranes and binds to cellular DNA, causing a shift in fluorescence intensity proportional to the extent of membrane damage. A representative heat‐killed PI‐positive control of *K. phaffii*, shown on the top histograms of Figure 5a, illustrates the fluorescent shift expected in unviable cells. Histograms of PI‐stained samples following methanol induction reveal multimodal peaks (Figure 5a). The rightmost peak overlapping with the PI‐positive control indicates the fraction of unviable cells at different time points throughout the perfusion processes. At the end of AP2 and MP2, 91.39% and 93.45% of the analyzed populations, respectively, can be considered viable. Similar viabilities (above 90%) have previously been achieved in *K. phaffii* fed‐batch cultivations,^64^, ^67^ whereas depending on the process conditions unviable populations of up to 30% can be observed.^65^, ^67^

The shift in Median Fluorescence Intensity (MFI) (Figure 5b) describes the impact of the processes on the entire population, accounting not only for unviable cells, but also for those with a different degree of membrane damage. For each analysis, a separate PI‐positive control was prepared to verify the activity of the dye at the specific time point. Change in membrane integrity is further calculated as relative MFI: the shift in fluorescence of PI‐stained samples relative to the PI‐positive control (Figure 5b). The relative MFI increases from 0.68% to 2.48% for MP2, and from 0.88% to 1.92% for AP2 after approximately 24 and 200 h of perfusion, respectively.

**FIGURE 6:**
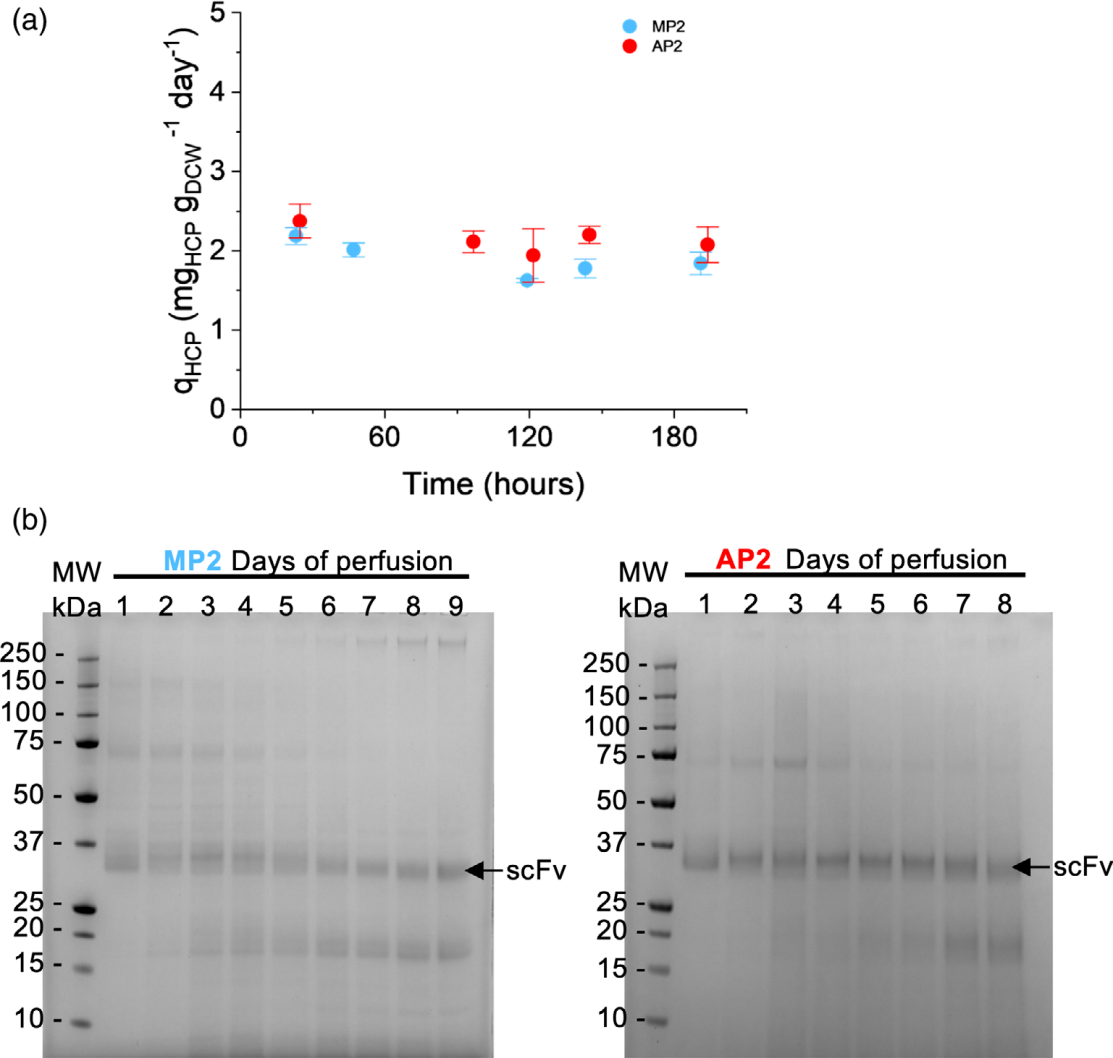
HCP analysis of MP2 (blue) and AP2 (red) perfusate samples. The indicated time corresponds to the hours or days of operation in perfusion‐based mode. (a) Specific production rates calculated from HCP concentrations (technical duplicates), obtained by ELISA analysis, and biomass concentration (technical triplicates) determined by dry cell measurements. Error bars represent the propagated error. (b) SDS‐PAGE analysis of perfusate samples from MP2 (left image), and AP2 (right image).

Collectively, the flow cytometry data indicate a low degree of cell damage, with a slow and similar decline of *K. phaffii*'s viability over time for the two processes (Figure S3). The quantification of host cell proteins (HCPs) indicated stable titers throughout the processes, thus supporting the flow cytometry findings (Figure 6a). Notably, the SDS‐PAGE analysis reveals a shift from high‐molecular to low‐molecular bands with the progression of both processes (Figure 6b). This could be explained by an increase in proteolytic activity or changes in protein expression due to metabolic shifts.

Although the impact of shear stress might be higher for the VMF device due to an additional recirculation through the membrane module and the oscillation of the device, we did not find strong evidence of device‐specific decline in cell viability for either of the tested technologies. Both systems were tested beyond the average duration of fed‐batch protein induction phase of ~4 days,^16^, ^64^, ^67^ signifying promising application in *K. phaffii* perfusion systems. Although membrane damage increases over time, the change occurs at a slow rate (Figure S3). Based on the change of relative MFI, we project a daily increase in membrane damage of 0.15% and 0.25% for the AP and MP processes, respectively (Figure S3A). When the fraction of unviable *K. phaffii* cells is used instead, a daily increase of 0.75% and 0.73% is expected for AP and MP processes, respectively (Figure S3B). In retrospect, extending the process duration even further, would have additionally revealed the cumulative impact of cell aging and cell retention techniques on the observed here trends.

### 3.4 Protein production

A multitude of factors influence yields in recombinant protein production. *K. phaffii*'s lower cell‐specific productivities are often mentioned as the main drawback when compared to mammalian cells.^39^ Due to the current advances in *K. phaffii* recombinant protein production and the increasing need for flexible biomanufacturing processes, the alternative host might become relevant for processes at different scales and throughputs. Improvements in *K. phaffii* processes are pursued and achieved through several approaches, such as strain engineering,^8^, ^68^, ^69^ methanol feeding strategies,^70^, ^71^ and optimizing process variables such as temperature,^72^ pH and media components.^73^ By examining whether and how the type of cell retention affects *K. phaffii*'s productivity in perfusion‐based processes, we highlight several important aspects that must be considered as this mode of operation evolves and extends to alternative hosts.

**FIGURE 7:**
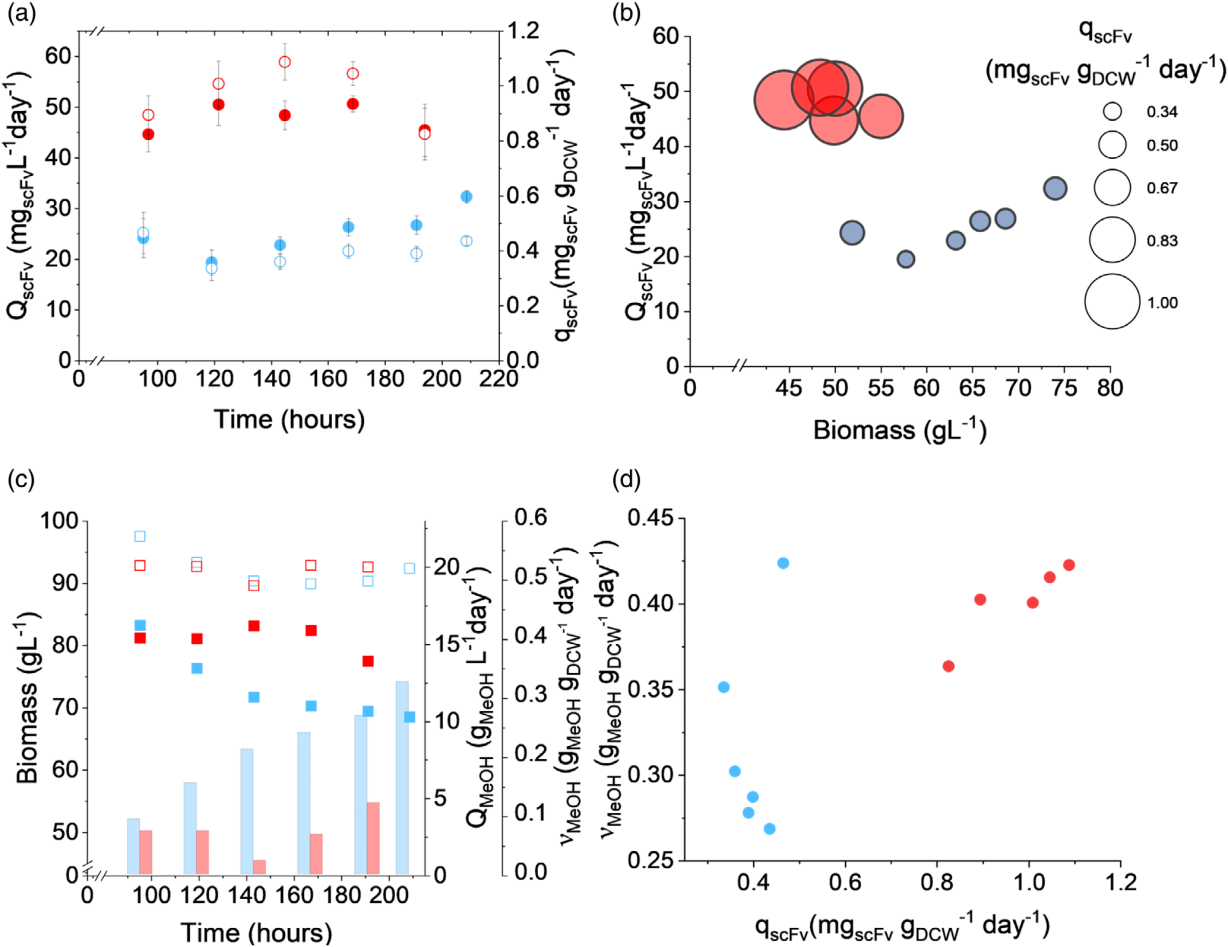
Productivity of acoustic and membrane perfusion‐based processes. Process MP2 is depicted in blue and process AP2 in red. (a) Time course of volumetric productivity, *Q*_scFv_, and specific productivity, *q*_scFv_, calculated from scFv13R4 concentration in harvest samples (technical triplicates) and biomass concentration (technical triplicates) measured by dry cell weight. *Q*_scFv_ is represented by closed, and *q*_scFv13R4_ by open symbols. Error bars represent the propagated errors. The indicated time corresponds to the hours of operation in perfusion‐based mode. (b) Bubble plot of biomass concentration and volumetric productivity, *Q*_scFv_, sized by the corresponding specific productivity, *q*_scFv_. Legend for bubble size, *q*_scFv_, is shown on the right of the panel. The mean values of *Q*_scFv_ and *q*_scFv_, shown in (a), and their corresponding biomass concentrations are used for illustrating the relationship. (c) Time course of volumetric methanol consumption rate, *Q*_MeOH_ (open symbols) and specific methanol consumption rate, $\nu_{\text{MeOH}}$ (closed symbols). The mean values of the corresponding biomass concentrations are shown as bars. (d) Relationship between cell‐specific productivity (*q*_scFv_) and the specific methanol consumption rate ($\nu_{\text{MEOH}}$) for the two processes.

The expression of scFv13R4 was sustained over the whole duration of the processes, without a significant loss in either volumetric or specific productivity (Figure 7a). Together with the stable HCP levels (Figure 6), these results demonstrate the absence of events that disrupt protein expression and are in alignment with the observed viability (Figure 5). Just as we have observed with respect to viability, extending the duration of the processes is feasible and beneficial for uncovering long‐term trends in productivity. These observations also highlight the need for small‐scale systems with improved automation and process control in order to facilitate more efficient and less labour‐intensive process development.^29^

When comparing the two processes, AP2 outperforms MP2 with respect to volumetric and specific productivity at different time points. MP2 productivity increases with time, whereas it remains steady for AP2 (Figure 7a). Therefore, the highest volumetric and specific productivity for MP2 is observed at the end of the process with values of 32.29 ± 1.21 mg L^−1^ day^−1^ and 0.44 ± 0.02 mg g^−1^ day^−1^, respectively. The highest measured volumetric and specific productivity for the AP2 process is 50.63 ± 1.63 mg L^−1^ day^−1^ and 1.09 ± 0.07 mg g^−1^ day^−1^, respectively. No significant change in volumetric productivity with the increase of biomass was observed for AP2 (Figure 7b), suggesting that the system has sufficient expression capacity to reach the maximum scFv13R4 production for the supplied methanol even at lower biomass concentrations. On the contrary, the increase of volumetric productivities with the increase of biomass is indicative that the maximum was not reached during MP2 (Figure 7b).

As filter‐based cell retention systems are inherently susceptible to product retention due to the presence of a physical barrier, we compared the scFv13R4 titer measured in the bioreactor and the harvest stream. The results demonstrate that product retention could not account for the observed reduced productivity in MP2 (Figure S4). Previous studies on perfusion bioreactors using ATF and TFF systems have attributed product loss primarily to membrane fouling at high biomass concentrations.^25^, ^74^ Our findings indicate that the dynamic component of the VMF mitigated membrane fouling, thereby also minimizing product loss due to retention.

The values of temperature, pH, and moving average DO levels (DO_movavg_) were within the same range for both processes (Figure S5). Although DO was controlled in the same manner, we observed fluctuations with a larger magnitude for MP processes in comparison to AP processes, despite the comparable levels of biomass concentration. Since DO_movavg_ was maintained above 20% for all processes, we expect that the fluctuations were not a contributing factor to the observed lower productivities for MP processes.

Perfusion processes are typically operated at a targeted biomass concentration. However, the processes described here were performed at accumulating biomass, with the aim of testing the highest cell density achievable with the two cell retention devices. Notably, a biomass‐to‐substrate yield of ~0.26 g_DCW_ g_MeOH_^−1^ was calculated for both processes. However, since cell bleed occurs via the harvest stream only in AP2, the retained cells in the two systems are likely to utilize methanol differently. This is further highlighted by the variation of cell size and complexity of *K. phaffii* in MP2 and AP2 (Figure 4), indicating unequal methanol allocation towards biomass formation and growth, including peroxisome biogenesis and biosynthesis of enzymes involved in methanol metabolism.

After approximately 100 h of methanol induction, biomass concentrations remained higher for MP2 in comparison to AP2 (Figures 2 and 7b). Since both processes were conducted under nearly identical cultivation conditions, the available methanol relative to the cell biomass was lower in MP2, resulting in reduced specific consumption rates despite comparable volumetric consumption rates (Figure 7c). Higher specific methanol consumption rates have been linked to stronger induction of P_*AOX1*_, increased peroxisome biogenesis, and higher levels of recombinant protein expression.^75^ As biomass increases in MP2, the overall energy demand for cellular maintenance is expected to exceed that of AP2, leaving less methanol available for growth and recombinant protein expression. Notably, as the cultures were strongly methanol‐limited, such differences in methanol utilization are directly reflected in the cell‐specific productivity observed for the two systems.

Even though the availability of methanol has directly contributed to the observed differences between the two processes, cell‐specific productivities were still significantly higher for AP2 at similar specific methanol consumption rates (Figure 7d). Therefore, our data suggest another factor must also influence the observed differences in productivity. Methanol utilization towards scFv13R4 expression appears to be more efficient in AP2 than in MP2, even when biomass concentrations and specific methanol consumption rates were the same, suggesting that the type of cell retention affects productivity. Cultures characterized by higher heterogeneity, such as MP2 (Figure 4), are likely to exhibit more diverse requirements for methanol and oxygen, with different cells metabolizing methanol at different rates. This can motivate the increased DO fluctuations during MP processes (Figure S5D), and the observed lower productivities (Figure 7). With a decrease in heterogeneity, as observed in the AP2 process (Figure 4), where larger and complex cells accumulate at the expense of small and less complex cells, a larger proportion of the supplied methanol is anticipated to be utilized towards the expression of the target protein, resulting in higher cell‐specific productivities (Figure 7a,b,d). Cells characterized by a large size and a higher complexity are expected to contain elevated levels of peroxisomes, rich in methanol utilization enzymes, thereby having an enhanced methanol utilization capacity and supporting higher protein production.^75^, ^76^, ^77^ Furthermore, as higher methanol fluxes are associated with greater peroxisome biogenesis, we hypothesize that the increase in specific methanol consumption rates in AP2 together with the selectivity of the ACS towards larger and more complex cells, had a cumulative effect on the superior performance of AP2.

Future studies focused on optimizing perfusion rates and methanol concentrations independently for the two systems are needed to fully demonstrate the potential of VMF and ACS in *K. phaffii* perfusion systems. For instance, too high perfusion rates might negatively impact the cell separation efficiency for the ACS‐based system as opposed to those using VMF for cell separation. Furthermore, due to the differences in cell heterogeneity observed here, the optimum cell‐specific perfusion rates (CSPR) for the two systems are likely to be different. Methanol utilization and the relationship between specific growth rate and P_*AOX1*_ regulation also have a significant impact on protein production kinetics and should be considered in the bioprocess design.^78^

## 4 CONCLUSIONS

Increasing the understanding of *K. phaffii* processes for recombinant protein production in perfusion mode is essential to harness the full potential of alternative hosts beyond the well‐established fed‐batch processes. This study demonstrates that both VMF and ACS have significant potential as cell retention devices in *K. phaffii* perfusion processes. Our results show that the superior performance of the acoustic‐based process largely stems from higher cell‐specific productivity, likely due to better utilization of the supplied methanol. Although lower cell‐specific productivity was observed for the membrane‐based process, our findings suggest that higher productivities could be achieved by conducting the process at higher biomass concentrations and by increasing the specific methanol consumption rate. Cell separation efficiencies of ~100% at biomass concentrations up to 74.1 ± 0.1 g L^−1^ were reached with the VMF technology used here, indicating that the limits of the device were not reached and are likely to be higher. The change in cell size and complexity of the retained biomass is the most prominent difference observed between the two processes and appears to be an important factor in determining productivities. Whereas ACS retains larger and more complex cells more efficiently, thus reducing the heterogeneity of the population, VMF retains all cells in the system. While further investigation is needed, these differences seem to be one of the primary reasons for the higher productivities in the acoustic‐based process. Cellular accumulation of AOX and other enzymes with major importance in methanol metabolism^56^, ^77^ is expected to cause the observed increase in complexity.

## AUTHOR CONTRIBUTIONS

**Marina Y. Linova:** conceptualization, data curation, formal analysis, investigation, methodology, visualization, writing **–** original draft, writing – review and editing. **Satish K. Kodiripaka:** investigation, methodology, writing – review and editing. **Edite Martins:** investigation, methodology, writing – review and editing. **Sobhana A. Sripada:** writing – review and editing. **Stefano Menegatti:** writing – review and editing. **John M. Woodley:** conceptualization, funding acquisition, writing – review and editing.

## CONFLICT OF INTEREST STATEMENT

The authors declare no conflict of interest.

## Supporting information


**Table S1.** Composition of BFM21 cultivation media and PTM4 trace mineral solution.
**Figure S1.** FSC‐SSC density plots at different times after the initiation of perfusion for AP and MP processes. P1 shows subpopulation characterized by small cell size and low complexity (low FSC and SSC), where P2 is characterized by larger cell size and higher complexity (higher FSC and SSC). Sampling time are shown in the upper left corner of each density plot.
**Figure S2.** Methanol quantification in collected perfusate for process AP2 (left) and MP2 (right). HPLC analysis confirms the depletion of methanol in experimental samples from AP2 and MP2. Representative standards at methanol concentration of 0.2% and 2.2%, and cultivation medium blank are also shown. The retention time of methanol is highlighted in yellow.
**Figure S3.** Time‐dependent changes of *K. phaffii* viability in AP2 (red) and MP2 (blue). The increase in (A) relative MFI and (B) fraction of unviable cells, both inversely correlated to viability, are used to describe the observed linear trends.
**Figure S4.** Comparison of scFv13R4 titer in the bioreactor (closed symbols) and in the perfusate stream (open symbols) for MP2. The indicated time corresponds to the hours of operation in perfusion mode. Error bars show standard deviations of technical triplicates.
**Figure S5.** Cultivation parameters during *K. phaffii* acoustic (AP1 and AP2) and membrane (MP1 and MP2) perfusion processes. The indicated time corresponds to the hours of operation in perfusion mode. (A) pH and temperature—all processes; (B) 3‐hour DO moving average – all processes; (C) DO, logged every 0.5 hours, for AP1 and AP2; (D) DO, logged every 0.5 hours, for MP1 and MP2.

## Data Availability

The data that support the findings of this study are available from the corresponding author upon reasonable request.
