# Supplementary material for: Effect of cell retention techniques in Komagataella phaffii lab‐scale continuous processes
Source: Biotechnol Prog. 2025 Nov 18;42(2):e70092. doi: 10.1002/btpr.70092 (PMC13055141; doi:10.1002/btpr.70092)
Supplement: Supplementary file 1 — Table S1. Composition of BFM21 cultivation media and PTM4 trace mineral solution. Figure S1. FSC‐SSC density plots at different times after the initiation of perfusion for AP and MP processes. P1 shows subpopulation characterized by small cell size and low complexity (low FSC and SSC), where P2 is characterized by larger cell size and higher complexity (higher FSC and SSC). Sampling time are shown in the upper left corner of each density plot. Figure S2. Methanol quantification in collected perfusate for process AP2 (left) and MP2 (right). HPLC analysis confirms the depletion of methanol in experimental samples from AP2 and MP2. Representative standards at methanol concentration of 0.2% and 2.2%, and cultivation medium blank are also shown. The retention time of methanol is highlighted in yellow. Figure S3. Time‐dependent changes of K. phaffii viability in AP2 (red) and MP2 (blue). The increase in (A) relative MFI and (B) fraction of unviable cells, both inversely correlated to viability, are used to describe the observed linear trends. Figure S4. Comparison of scFv13R4 titer in the bioreactor (closed symbols) and in the perfusate stream (open symbols) for MP2. The indicated time corresponds to the hours of operation in perfusion mode. Error bars show standard deviations of technical triplicates. Figure S5. Cultivation parameters during K. phaffii acoustic (AP1 and AP2) and membrane (MP1 and MP2) perfusion processes. The indicated time corresponds to the hours of operation in perfusion mode. (A) pH and temperature—all processes; (B) 3‐hour DO moving average – all processes; (C) DO, logged every 0.5 hours, for AP1 and AP2; (D) DO, logged every 0.5 hours, for MP1 and MP2. [file BTPR-42-e70092-s001.docx]

**Supporting information: Effect of cell retention techniques in *Komagataella phaffii lab-scale* continuous processes**

Marina Y. Linova^1^, Satish K. Kodiripaka^1^, Edite Martins^1^, Sobhana A. Sripada^2^, Stefano Menegatti^2,3^, John M. Woodley^1*^

# ^1^Department of Chemical and Biochemical Engineering, Technical University of Denmark, Kongens Lyngby, Denmark

^2^Department of Chemical and Biomolecular Engineering, North Carolina State University, North Carolina, USA

^3^Biomanufacturing Training and Education Center (BTEC), Raleigh, North Carolina, USA

*Corresponding author: jw@kt.dtu.dk

**Table S1.** Composition of BFM21 cultivation media and PTM4 trace mineral solution.

| **BFM21** | | |
| --- | --- | --- |
| Phosphoric Acid (85%) | H_3_PO_4_ | 3.5 mL/L |
| Calcium Sulfate | CaSO_4_ | 0.119 g/L |
| Potassium Sulfate | K_2_SO_4_ | 2.4 g/L |
| Magnesium Sulfate Heptahydrate | MgSO_4._7H_2_O | 1.95 g/L |
| Potassium Hydroxide | KOH | 0.65 g/L |
| Sodium Citrate | C_6_H_5_Na_3_O_7_ | 2.94 g/L |
| Ammonium Sulfate | (NH_4_)_2_SO_4_ | 5 g/L |
| Sodium Hydroxide | NaOH | 1.537 g/L |
| 1 M Acetate Buffer |  | 40 mL/L |
| **PTM4** | | |
| Sulfuric Acid | H_2_SO_4_ | 5.1 mL/L |
| Copper(II) Sulfate Pentahydrate | CuSO4.5H2O | 2 g/L |
| Sodium Iodide | NaI | 0.08 g/L |
| Manganese(II) Sulfate | MnSO_4_ | 3 g/L |
| Sodium Molybdate Dihydrate | Na_2_MoO4.2H_2_0 | 0.2 g/L |
| Boric Acid | H_3_BO_3_ | 0.02 g/L |
| Cobalt(II) Chloride | CoCl_2_ | 0.5 g/L |
| Zinc Chloride | ZnCl_2_ | 6.7 g/L |
| Iron(II) Sulfate | FeSO_4_ | 21.6 g/L |
| Biotin | C_10_H_16_N_2_O_3_S | 0.2 g/L |

**
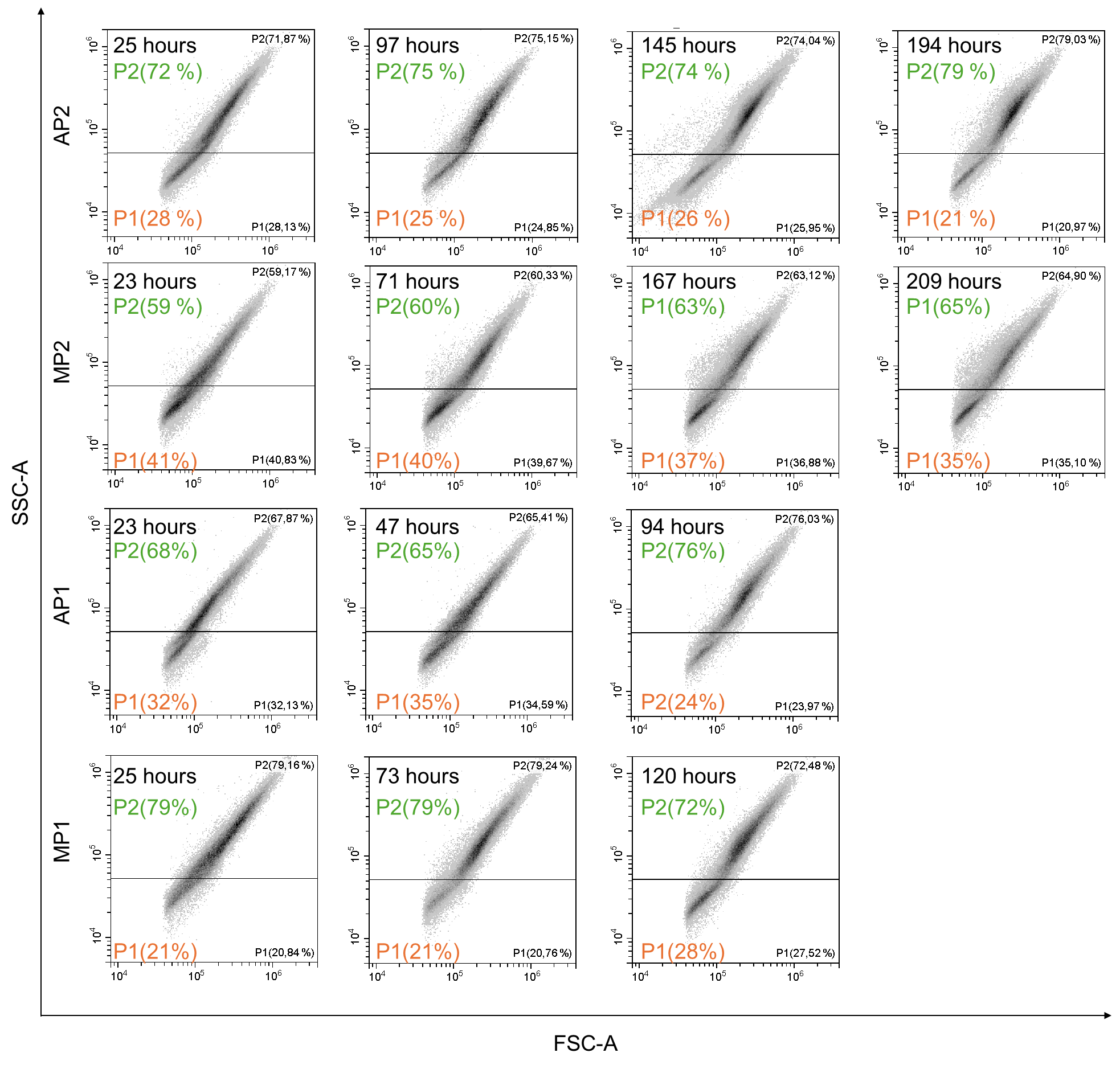
**

**Figure S1. FSC-SSC density plots at different times after the initiation of perfusion for AP and MP processes.** P1 shows subpopulation characterized by small cell size and low complexity (low FSC and SSC), where P2 is characterized by larger cell size and higher complexity (higher FSC and SSC). Sampling time are shown in the upper left corner of each density plot.

MP2

AP2


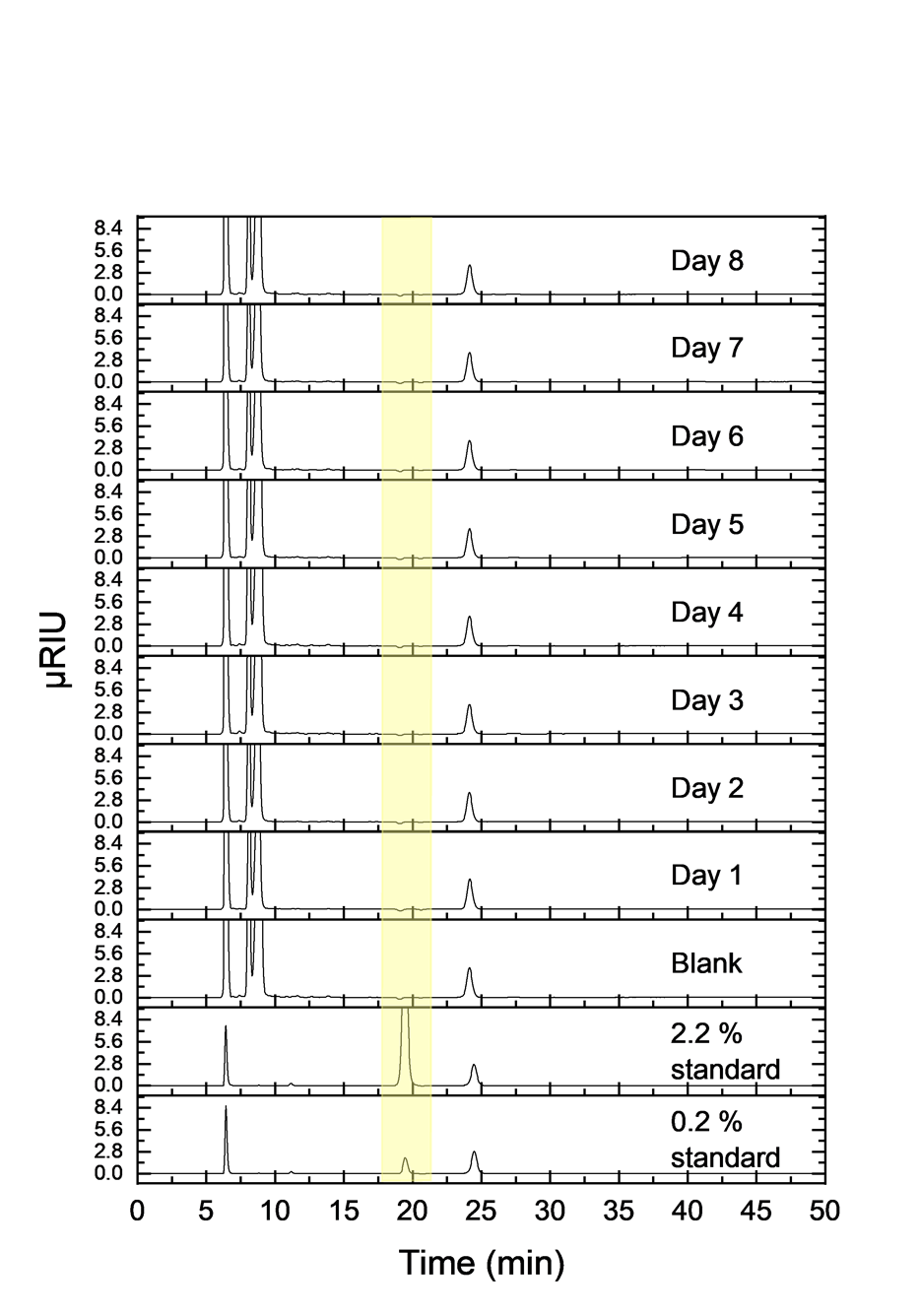

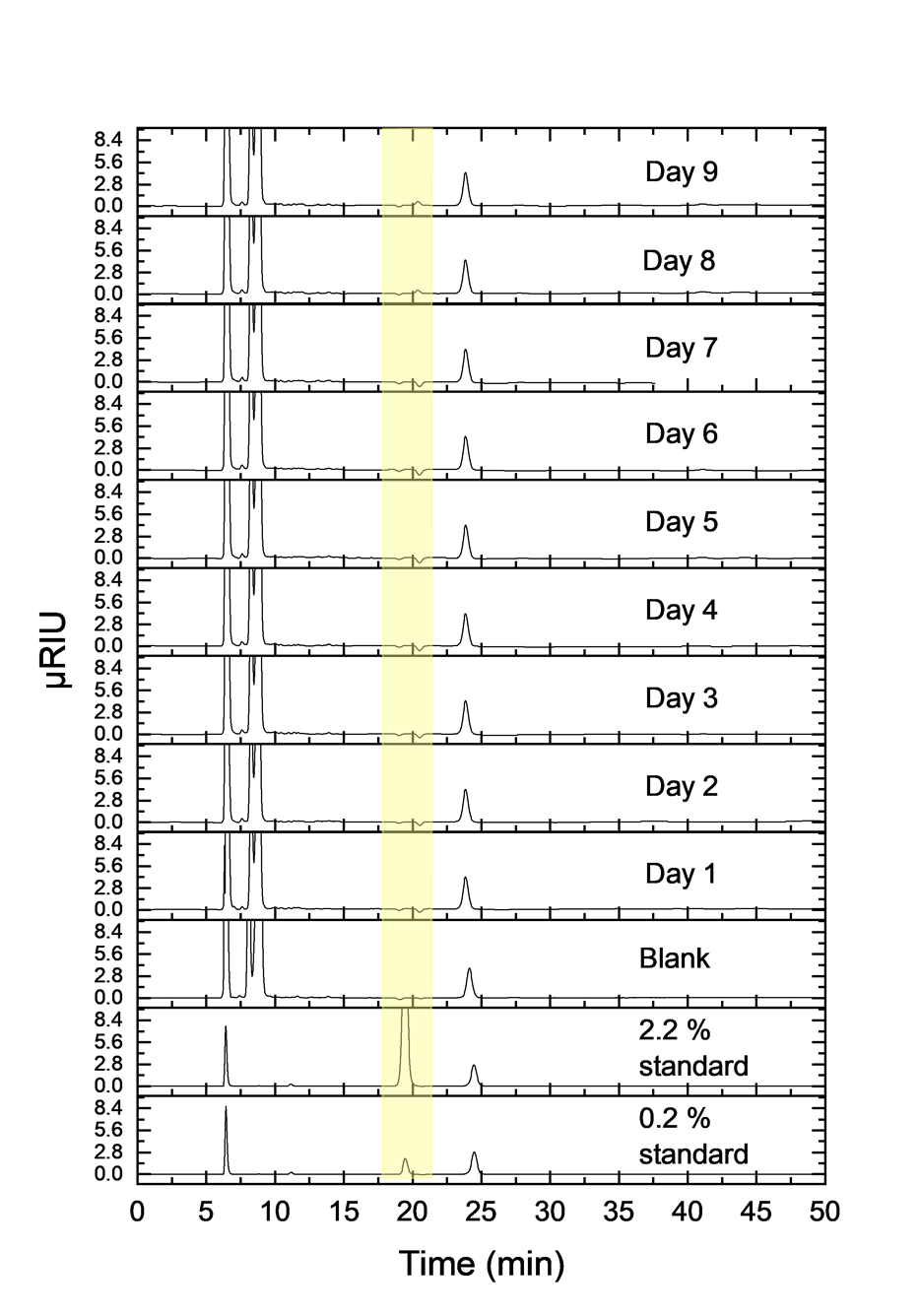


**Figure S2. Methanol quantification in collected perfusate for process AP2 (left) and MP2 (right).** HPLC analysis confirms the depletion of methanol in experimental samples from AP2 and MP2. Representative standards at methanol concentration of 0.2 % and 2.2 %, and cultivation medium blank are also shown. The retention time of methanol is highlighted in yellow.

**
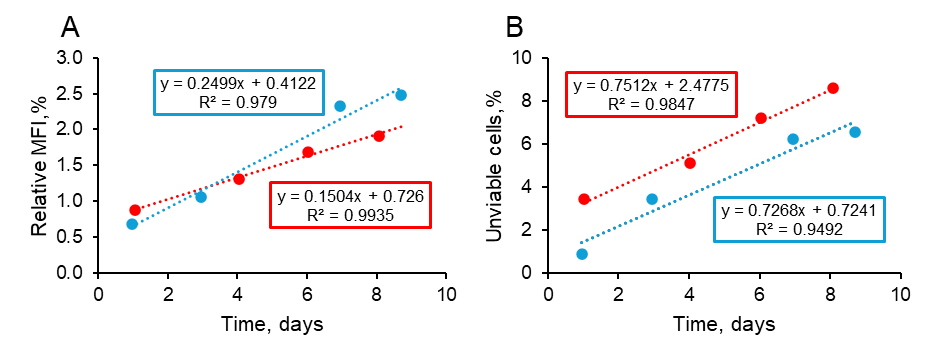
**

**Figure S3. Time-dependent changes of *K. phaffii* viability in AP2 (red) and MP2 (blue).** The increase in A) relative MFI and B) fraction of unviable cells, both inversely correlated to viability, are used to describe the observed linear trends.

**
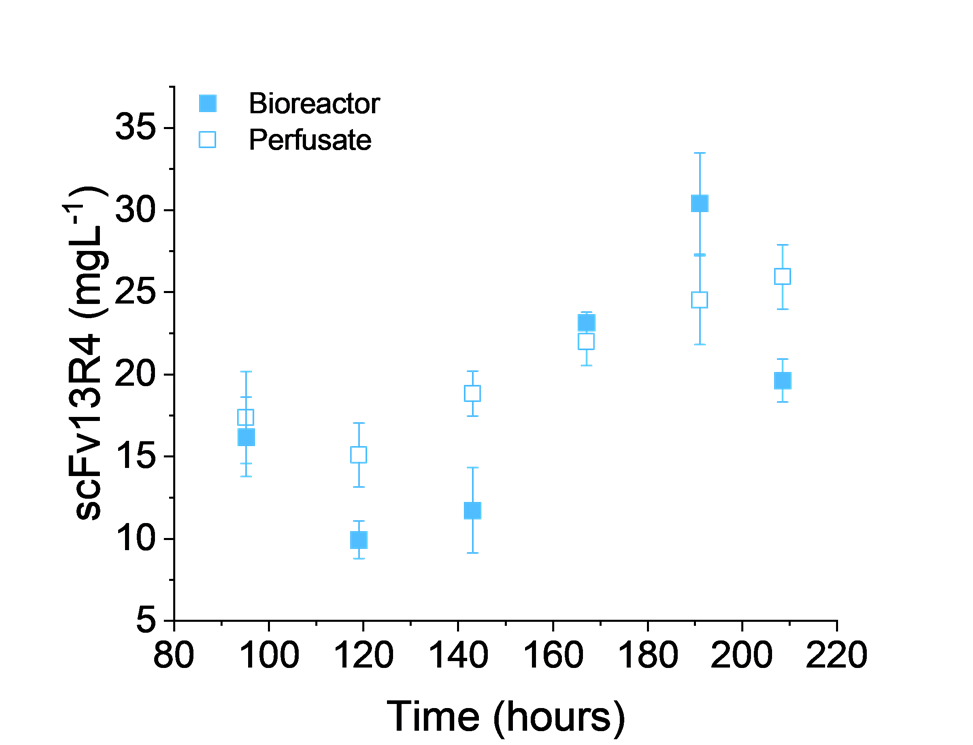
**

**Figure S4.** Comparison of scFv13R4 titer in the bioreactor (closed symbols) and in the perfusate stream (open symbols) for MP2. The indicated time corresponds to the hours of operation in perfusion mode. Error bars show standard deviations of technical triplicates.

**
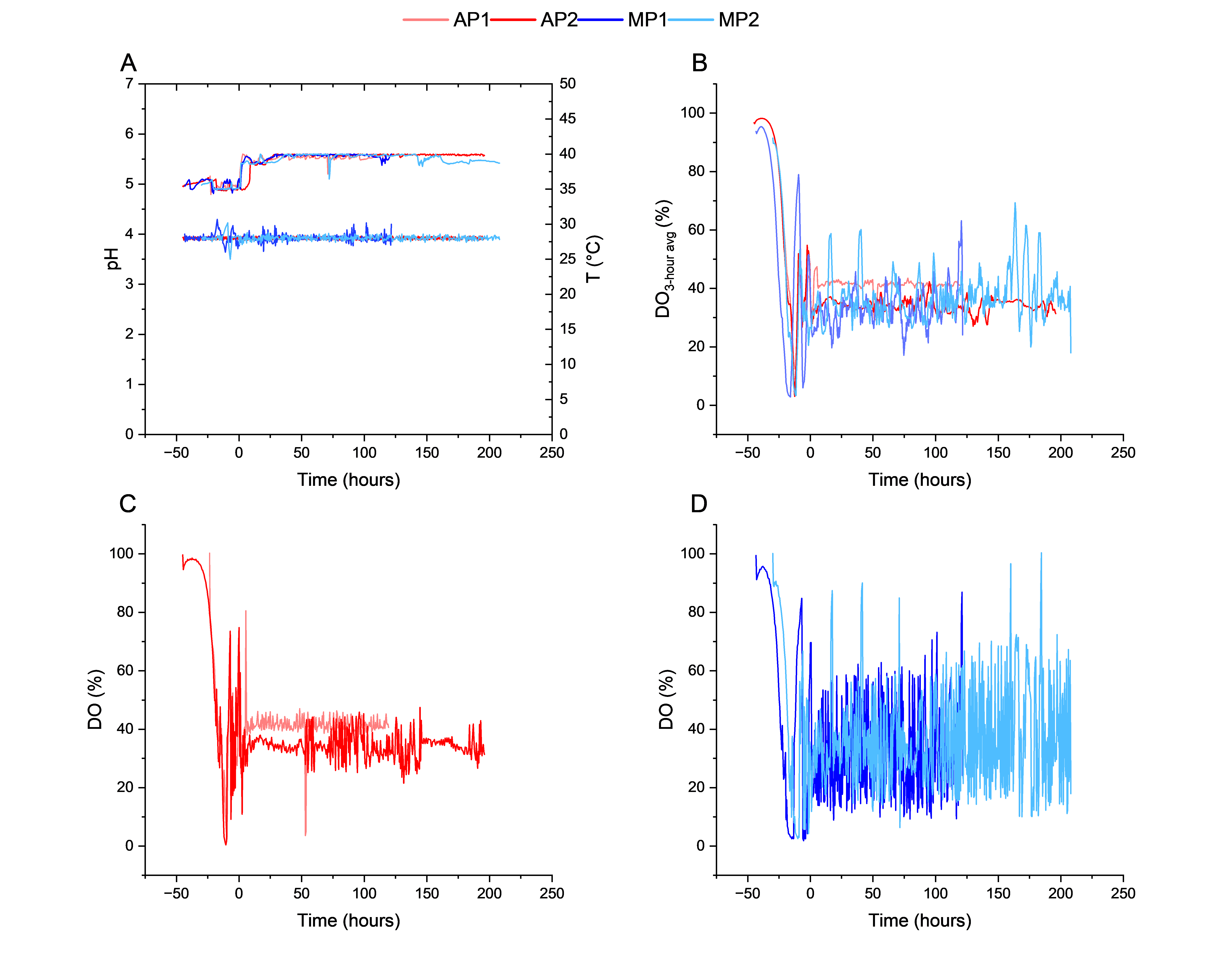
**

**Figure S5.** Cultivation parameters during *K.phaffii* acoustic (AP1 and AP2) and membrane (MP1 and MP2) perfusion processes. The indicated time corresponds to the hours of operation in perfusion mode. **A)** pH and temperature – all processes; **B)** 3-hour DO moving average – all processes; **C)** DO, logged every 0.5 hours, for AP1 and AP2; **D)** DO, logged every 0.5 hours, for MP1 and MP2**.**
